# An inherited genetic variant of the CEP72 gene is associated with the development of vincristine-induced peripheral neuropathy in female patients with aggressive B-cell lymphoma

**DOI:** 10.1007/s00277-024-05973-9

**Published:** 2024-09-04

**Authors:** Konstantinos Christofyllakis, Dominic Kaddu-Mulindwa, Vadim Lesan, Torben Rixecker, Igor Age Kos, Gerhard Held, Evi Regitz, Michael Pfreundschuh, Joerg Thomas Bittenbring, Lorenz Thurner, Viola Poeschel, Marita Ziepert, Bettina Altmann, Moritz Bewarder

**Affiliations:** 1 Department of Internal Medicine I (Oncology, Hematology, Clinical Immunology, and Rheumatology), Saarland University Medical Center, Homburg/Saar, Germany; 2 Institute for Medical Informatics, Statistics and Epidemiology, Leipzig University, Leipzig, Germany

**Keywords:** Vincristine, Polyneuropathy, Aggressive lymphoma, Single nucleotide polymorphism, DLBCL, Pharmacogenomics

## Abstract

Vincristine-induced peripheral neuropathy (VIPN) is an adverse effect of regimens used for the treatment of aggressive B-cell non-Hodgkin lymphoma (B-NHL). A single-nucleotide polymorphism (SNP) in the promotor region of the *CEP72* gene has been identified as risk factor for the development of VIPN in children. To validate these results in adults we aimed to determine the association of the high-risk *CEP72* (rs924607 TT genotype) with the occurrence and severity of VIPN. Analysis of SNP rs924607 (TT, CC or CT) was performed in all enrolled patients with available blood samples with a TaqMan genotyping assay. Rates and grades of VIPN were assessed prospectively as part of the RICOVER-60 trial. *CEP72* genotype could be assessed in 519 patients. VIPN data was available for 499/519 patients who were included in the final analysis. 286 (57%) patients developed VIPN of any grade during treatment. Grade 2–4 VIPN occurred in 33% (166/499) of patients. The high-risk *CEP72* TT genotype at rs924607 was identified in 97/499 (19%) patients. The TT genotype was not correlated with VIPN in the overall study population compared to patients with either CC or CT genotypes (*p* = 0.748). However, in the subgroup of female patients, the TT genotype was associated with increased occurrence of any-grade VIPN as well as grade 2–4 VIPN as compared to patients with either CC or CT genotypes (*p* = 0.016 and *p* = 0.020, respectively). Thus, the SNP rs924607 in the *CEP72* gene is associated with increased VIPN incidence in female patients with aggressive B-NHL treated with CHOP chemotherapy. Trial registration ClinicalTrials.gov identifier: NCT00052936, submission date: 2005-06-23, EudraCT Number: 2010-019587-36.

**Supplementary Information:**

The online version contains supplementary material available at 10.1007/s00277-024-05973-9.

## Introduction

Diffuse large B-cell lymphoma (DLBCL) is the most common aggressive B-cell non-Hodgkin lymphoma (B-NHL) accounting for 25–35% of all NHL cases in developed countries and even more in developing countries [1]. ^,^ [2] The R-CHOP protocol (rituximab, cyclophosphamide, doxorubicin, vincristine (VCR), prednisone) is the current standard of care in patients with DLBCL in first-line therapy [3]. ^,^ [4]^,^ [5] Recently, the replacement of VCR with polatuzumab vedotin (pola-R-CHP) showed improved progression-free survival (PFS) rates in patients with newly diagnosed DLBCL representing an additional therapeutic option in the first-line setting [6]. Due to high cure rates of DLBCL with long term survival rates of 70–80% in young patients with good prognosis [3], treatment-related adverse events such as chemotherapy-induced polyneuropathy (CIPN) are becoming increasingly apparent. In an effort to reduce chemotherapy-induced side effects, the recent FLYER trial compared 6 versus 4 cycles of R-CHOP in young, good risk DLBCL patients [7]. Using the (R-)CHOP protocol, VCR is the main cause for the development of polyneuropathy (PNP), which led to the description of vincristine induced polyneuropathy (VIPN). More than 20% of patients receiving (R-)CHOP with or without rituximab are going to develop VIPN [8]. ^,^ [4] It is characterized by loss of motor function, sensory dysfunction and neuropathic pain causing considerable morbidity and affecting the quality of life of patients even though the underlying mechanisms of VIPN are still not clear [9]. ^,^ [10]^,^ [11]^,^ [12] Vincristine, a vinca alkaloid, targets mitotic-spindle microtubules rendering replicating cells unable to separate their chromosomes and therefore inducing apoptosis [13]. ^,^ [14]^,^ [9]^,^ [10] In neurons, vinca alkaloids destabilize microtubule formation, which leads to impairment of axonal transport and mitochondria function [9]. It has been shown, that the incidence of VIPN is associated with the cumulative dose of vincristine and ethnicity [15]. ^,^ [16] In the RICOVER-60 trial VCR dose reductions due to PNP were observed more often in female patients (patients receiving full dose vincristine: male 60%, female 40%, *p* < 0.001). However, VCR dose reductions had no impact on survival outcomes [17]. Therapeutic options for symptomatic patients are scarce ranging from treatment discontinuation and dose reductions to the application of antidepressant or anticonvulsive drugs like duloxetine, pregabalin and gabapentin [18]. In an attempt to determine genetic risk factors for developing VIPN, genome-wide association studies (GWAS) have been used to identify single-nucleotide polymorphisms (SNPs) associated with VIPN. These studies were performed mainly in children receiving polychemotherapy including vincristine for acute lymphoblastic leukemia (ALL) [19]. ^,^ [20]^,^ [21]^,^ [22] Diouf and colleagues reported a strong association between an inherited SNP in the promoter region of the gene *CEP72*, (rs924607, C > T), with an increased risk for and severity of developing VIPN [19]. *CEP72* encodes a centrosomal protein that is involved in microtubule formation. In patients homozygous for the risk allele (TT), the cumulative incidence of neuropathy and the mean grade of neuropathy were significantly higher than in patients with a CT or CC genotype. Since this original report, another retrospective study including adults receiving treatment for ALL and a meta-analysis could confirm the association of the respective SNP with VIPN [20]. ^,^ [23] In contrast to this, two studies including children treated for ALL, found no association of the rs924607 SNP with VIPN [21]. ^,^ [22] In other entities this association is far less studied and for B-cell lymphoma only one small retrospective Japanese study, which included patients treated with R-CHOP at a single center, exists [24]. This study could not find an association between VIPN and homozygosity for the high risk *CEP72* genotype (TT). However, due to the small number of patients included (*n* = 56) weaker associations might have been overlooked.

To determine whether the SNP rs924607 is associated with VIPN in adult patients with aggressive B-cell lymphoma and to investigate possible sex differences in VIPN development we performed CEP72 SNP rs924607 genotyping in 519 patients treated within the RICOVER-60 trial of the German High-Grade Non-Hodgkin Lymphoma Study Group (DSHNHL) / German Lymphoma Alliance (GLA) receiving R-CHOP and analyzed the association of CEP72 genotype with the incidence and severity of VIPN and the influence of sex in this context [25]. 

## Patients and methods

### Study population

We performed a retrospective analysis of the prospective RICOVER-60 trial. All patients with available germinal DNA for SNP analysis and available data on neuropathy were included in the analysis set. In the RICOVER-60 trial (NCT00052936) 1222 elderly patients aged 61–80 years with newly diagnosed CD20 positive aggressive B-cell lymphoma were included to receive 6 or 8 cycles of CHOP chemotherapy every 2 weeks with or without rituximab. Radiotherapy was planned to sites of initial bulky disease with or without extranodal involvement. Vincristine was administered at a dose of 1.4 mg/m^2^ with a maximum dose of 2 mg. Adverse events were monitored and documented as part of the required clinical trial adverse event monitoring and patients were assessed for the presence of neuropathy by the treating physician at each visit during treatment. Rates and grades of neuropathy were assessed according to the National Cancer Institute (NCI) Common Terminology Criteria for Adverse Events (CTCAE) version 4.3. The RICOVER-60 trial reported the rates of “neuropathy” and not specifically VIPN. However, it can be assumed, that all neuropathy reported is VIPN, thus, the latter term is used in this manuscript to better align with the relevant literature. This study was approved by the ethical review committee of each participating centre. The study was performed in accordance with the rules of the Declaration of Helsinki after obtaining written consent from the patients.

### Genotyping

Genomic DNA was isolated from peripheral whole blood using the QIAamp DNA Blood Kit (QIAGEN QIAamp, Hilden, Germany). The SNP of interest (SNP Database ID: rs924607), located within the promoter region of the CEP72 gene on chromosome 5p15.33, was analyzed using a TaqMan genotyping assay^®^ (Assay ID C__8292459_20; Applied Biosystems, Foster City, CA, USA). The DNA was diluted in water to a final concentration of 5 ng/µL. A total of 2.25 µL diluted genomic DNA was mixed with 2.5 µL TaqMan Universal Master Mix and 0.25 µL TaqMan SNP Genotyping Assay Mix. Reactions were performed according to the following protocol: Denaturation (95 °C, 10 min (min)), followed by 45 cycles of denaturation (92 °C, 15 s), annealing (60 °C, 1 min) and extension (60 °C, 1 min). The polymerase chain reaction (PCR) was performed using the Real-Time PCR system StepOnePlus (Invitrogen; Thermo Fisher Scientific, Inc.). To discern alleles, fluorescence was measured post-PCR at a temperature of 60 °C for 1 min using the cfx manager software version 3.1.1517 (Bio-Rad Laboratories, Inc.).

### Statistical analysis

For comparison of patient characteristics, chi-square and, if necessary, Fisher’s exact tests were used. For comparison of the median age, Mann-Whitney U test was used. Test of the Hardy Weinberg equilibrium was used to assess allele distribution of CEP72 rs924607. Vincristine-induced peripheral neuropathy (grade 0 versus 1–4/2–4/3–4) was compared for 6 versus 8 cycles of CHOP-14 ± 8xR, for male versus female patients (within all, CC/CT and TT genotype) and for CC/TT versus TT genotype (within all, male, female patients) using chi-square and, if necessary, Fisher’s exact test. In addition, VIPN was analyzed using univariate and multivariate logistic regression models including an interaction term. Odds ratio (OR) with 95% Confidence Interval (CI) were presented. The two-sided significance level was *p* < 0.050. No adjustments were made for multiple comparisons. Statistical analyses were performed with IBM SPSS Statistics 26 and 29 software (SPSS, Chicago, IL). Figures were generated using GraphPad Prism version 10.2.2 (GraphPad Software, Boston, MA).

## Results

### Patient characteristics

Germline DNA for SNP analysis was available in 519 patients with aggressive B-cell lymphoma of the total 1222 patients. Of those, data on VIPN was available for 499 patients, which represented our final study population (Figure S1). Demographic and clinical characteristics as well as distribution of CEP72 rs924607 genotype of all patients are summarized in Table 1. Regarding CEP72 rs924607 genotype, 97/499 (19%) were homozygous for the high-risk TT genotype, 252/499 (51%) were heterozygous (CT genotype) and 150/499 (30%) were homozygous for the CC genotype. SNP analysis and vincristine-induced peripheral neuropathy were assessed in 499 patients of whom 235 (47%) were female. The baseline characteristics of the 499 patients with available germline DNA and documented VIPN who were analyzed were compared to all other patients in the study, showing no relevant statistical differences between the analyzed cohort and the not analyzed population (Table S1). Of 499 patients, 248 (50%) received six cycles of CHOP with or without rituximab and 251 (50%) received eight cycles of CHOP with or without rituximab.

**Table 1:** Demographics and clinical characteristics of all analyzed patients

	Male (*n* = 264)		Female (*n* = 235)		Total (*n* = 499)	
	CC/CT (*n* = 211)	TT (*n* = 53)	CC/CT (*n* = 191)	TT (*n* = 44)	CC/CT (*n* = 402)	TT (*n* = 97)
Age, median (range)	67 (61, 80)	68 (61, 78)	69 (61, 80)	70 (61, 78)	68 (61, 80)	69 (61, 80)
LDH > UNV	90 (43%)	23 (43%)	103 (54%)	23 (52%)	193 (48%)	46 (47%)
ECOG > 1	23 (11%)	3 (6%)	31 (16%)	6 (14%)	54 (13%)	9 (9%)
Stage III/ IV	107 (51%)	23 (43%)	100 (52%)	25 (57%)	207 (51%)	48 (49%)
Extralymph. involvement	130 (62%)	24 (45%)	91 (48%)	23 (52%)	221 (55%)	47 (48%)
Extralymph. involvement > 1	39 (18%)	5 (9%)	31 (16%)	9 (20%)	70 (17%)	14 (14%)
IPI 1 2 3 4,5	76 (36%) 47 (22%) 59 (28%) 29 (14%)	21 (40%) 16 (30%) 11 (21%) 5 (9%)	48 (25%) 63 (33%) 45 (24%) 35 (18%)	13 (30%) 10 (23%) 12 (27%) 9 (20%)	124 (31%) 110 (27%) 104 (26%) 64 (16%)	34 (35%) 26 (27%) 23 (24%) 14 (14%)
Bulky disease	81 (38%)	19 (36%)	85 (45%)	14 (32%)	166 (41%)	33 (34%)
B symptoms	62 (29%)	20 (38%)	68 (36%)	16 (36%)	130 (32%)	36 (37%)
BM involvement	7 (3%)	3 (6%)	13 (7%)	3 (7%)	20 (5%)	6 (6%)
Reference pathology* DLBCL other B-cell other	171 (81%) 38 (18%) 1 (0.5%)	42 (81%) 10 (19%) 0 (0%)	159 (83%) 30 (16%) 2 (1%)	35 (80%) 9 (20%) 0 (0%)	330 (82%) 68 (17%) 3 (1%)	77 (80%) 19 (20%) 0 (0%)
6 x CHOP-14 ± 8 x R 8 x CHOP-14 ± 8 x R	104 (49%) 107 (51%)	27 (51%) 26 (49%)	95 (50%) 96 (50%)	22 (50%) 22 (50%)	199 (50%) 203 (50%)	49 (50%) 48 (50%)

*Abbreviations* LDH = Lactatdehydrogenase; UNV = Upper Normal Value, ECOG = Eastern Cooperative Oncology Group performance status, extralymph. = extralymphatic; inv. = involvement; BM = bone marrow, DLBCL = diffuse large B-cell lymphoma

*some missing values

### Vincristine-induced peripheral neuropathy

**Fig. 1:**
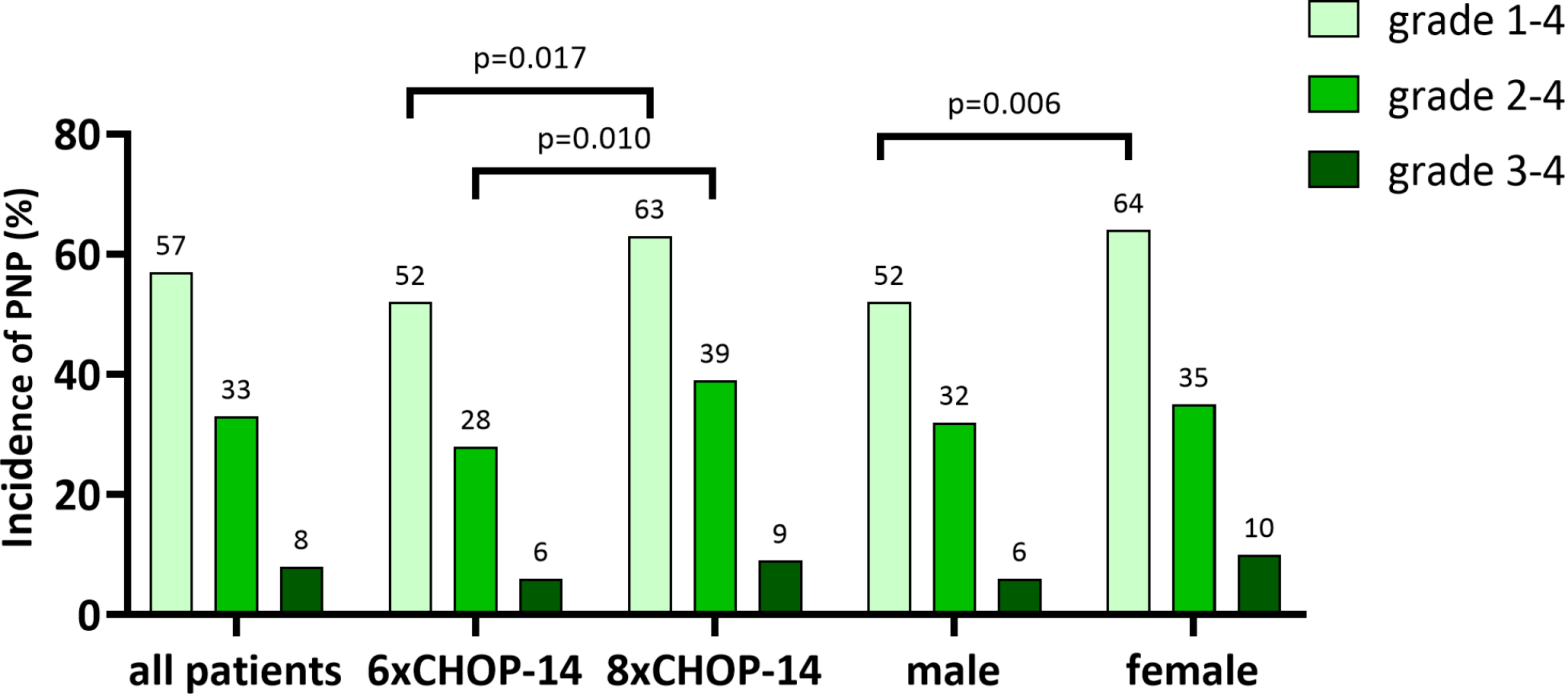
Incidence of PNP in the study population. Light green bars represent the percentage of patients with grade 1–4 PNP, medium shade green bars represent the percentage of patients with grade 2–4 PNP, and dark green bars represent the percentage of patients with grade 3–4 PNP. Incidence of PNP is shown for all patients, patients who received 6 cycles of CHOP-14 ± R, patients who received 8 cycles of CHOP-14 ± R, male and female patients. Statistically significant p values are shown

Vincristine-induced peripheral neuropathy of any grade during chemotherapy occurred in 286/499 (57%) patients (Fig. 1). In detail, 157/251 (63%) patients receiving eight cycles of chemotherapy developed VIPN of any grade compared to only 129/248 (52%) patients receiving six cycles of chemotherapy (*p* = 0.017). Female patients reported higher incidence of VIPN grade 1–4 than male patients (64% vs. 52%, *p* = 0.006). Grade 2–4 VIPN occurred in 166/499 (33%) patients. Eight cycles of chemotherapy resulted in higher incidence of grade 2–4 VIPN compared to six cycles of chemotherapy (39% vs. 28%, *p* = 0.010). Grade 2–4 VIPN occurred in 82/235 (35%) females and 84/264 (32%) male patients. Severe (grade 3–4) VIPN was reported in 38/499 (8%) patients. Patients receiving 8 cycles of CHOP developed grade 3–4 VIPN more often than patients receiving 6 cycles (9% vs. 6%, *p* = 0.238). Moreover, female patients developed severe VIPN more often than male patients (10% versus 6%, *p* = 0.084).

### Association of CEP72 rs924607 genotype with incidence and severity of VIPN

**Fig. 2:**
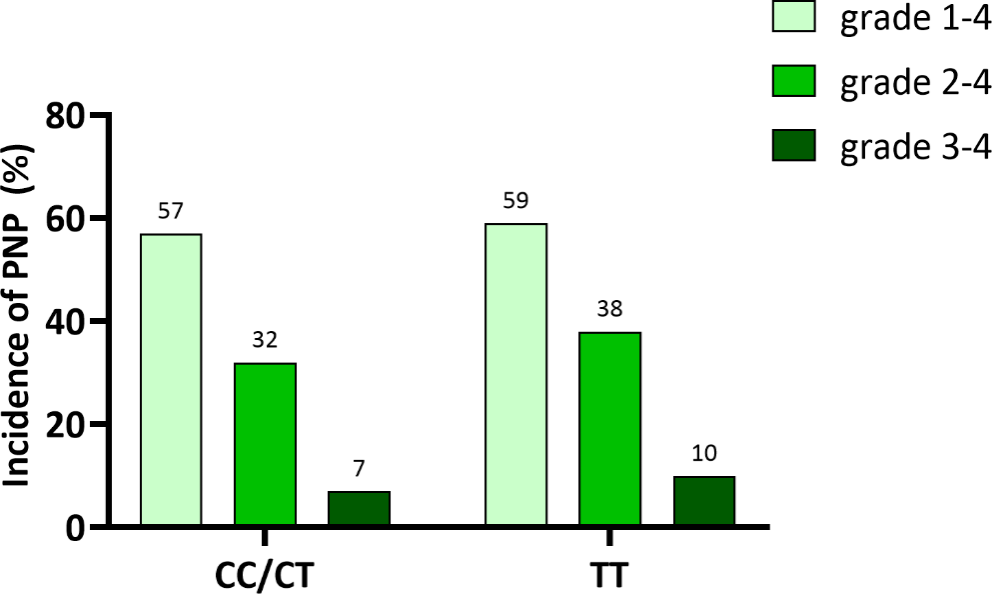
Incidence of PNP according to the presence of homozygosity of rs924607 SNP. Light green bars represent the percentage of patients with grade 1–4 PNP, medium shade green bars represent the percentage of patients with grade 2–4 PNP, and dark green bars represent the percentage of patients with grade 3–4 PNP. Incidence of PNP is shown for all patients who are homozygous for the rs924607 SNP (right) as opposed to all other patients (left). No statistically significant p-values were observed

Grade 1–4 VIPN occurred in 57/97 (59%) patients with the high-risk CEP72 TT genotype and in 229/402 (57%) patients with CC or CT genotype (CC/CT). This difference was not statistically significant (*p* = 0.748) (Fig. 2). Even after breaking down the incidence of VIPN within the individual genotypes, no statistically significant difference could be shown [CT genotype: 136/252 (54%), CC genotype: 93/150 (62%), *p* = 0.275) (Figure S2). Patients with high-risk TT genotype had only numerically higher incidence of VIPN grade 2–4 and VIPN grade 3–4 compared to CC/CT genotype without reaching statistical significance (VIPN grade 2–4: *p* = 0.256; VIPN grade 3–4: *p* = 0.265) (Fig. 2). Similarly, no statistically significant difference was observed between TT and the CC and CT genotypes separately (Figure S2).

**Fig. 3:**
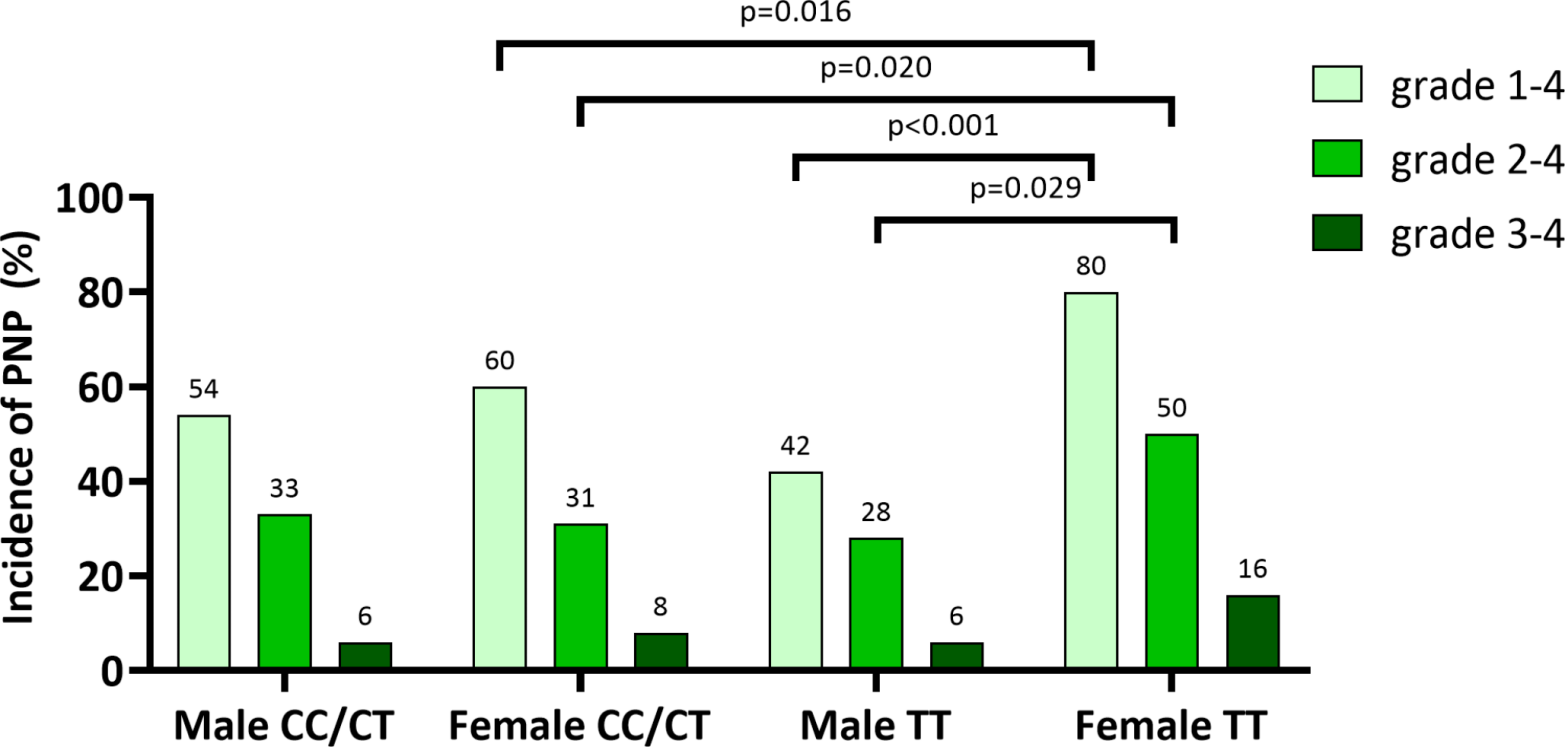
Sex-specific incidence of PNP according to the presence of homozygosity of rs924607 SNP. Light green bars represent the percentage of patients with grade 1–4 PNP, medium shade green bars represent the percentage of patients with grade 2–4 PNP, and dark green bars represent the percentage of patients with grade 3–4 PNP. Incidence of PNP is shown for male patients who are homozygous for the rs924607 SNP (middle right), male patients without homozygosity for the rs924607 SNP (left), female patients who are homozygous for the rs924607 SNP (right), female patients without homozygosity for the rs924607 SNP (middle left). Statistically significant p values are shown

However, higher rates of neuropathy were shown for females. Grade 1–4 VIPN incidence in female patients with TT genotype was significantly higher compared to male patients with TT genotype ((35/44 (80%) vs. 22/53 (42%), *p* < 0.001) (Fig. 3). More female patients with TT genotype developed grade 2–4 VIPN than male patients with TT genotype (22/44 (50%) vs. 15/53 (28%), *p* = 0.029).

In the sex-specific analysis, the impact of the high-risk genotype became clear. Specifically, female patients with high-risk TT genotype had a higher incidence of all-grade VIPN compared to female patients with CC and CT genotypes (TT: 35/44 (80%) vs. CC/CT: 115/191 (60%), *p* = 0.016) (Fig. 3). The Odds Ratio (OR) of developing all grade VIPN in females with TT genotype compared to females with CC/CT genotype was 2.6 (95%CI: 1.2–5.7, *p* = 0.019). In addition, females with TT genotype experienced more frequently grade 2–4 VIPN compared to females with CC/CT genotype (TT: 22/44 (50%) vs. 60/191 (31%), p-value = 0.020) (Fig. 3). The OR of developing grade 2–4 VIPN in females with TT genotype compared to females with CC/CT genotype was 2.2 (95%CI: 1.1–4.2, *p* = 0.021).

In the subgroup of female patients, 7/44 (16%) with TT genotype developed grade 3–4 VIPN as compared to 16/191 (8%) with CC/CT genotype not reaching statistical significance (*p* = 0.157) (Fig. 3). VIPN incidence in all genotypes according to sex is shown in Figure S3.

In the multivariate logistic regression model for VIPN grade 1–4 including sex, genotype and the interaction term (sex*genotype) a significant interaction between sex (female versus male) and genotype (TT versus TT/CT) was found (OR for the interaction: 4.3, 95% CI 1.6–11.5, *p* = 0.004). Further adjustment for 6 or 8 cycles CHOP or for body surface area (BSA) in logistic regression model did not change this result.

## Discussion

With high cure rates, treatment related toxicities remain an unsolved issue in clinical outcomes of adult patients with aggressive B-cell lymphoma, thus making identification of risk factors essential to pave the way for preventive strategies. Several studies aimed to confirm the association of the SNP rs924607 with VIPN in adults but yielded conflicting results, which may in part be attributed to the small number of patients studied [20]. ^,^ [24] We therefore analyzed 499 patients who were treated within the phase III RICOVER-60 trial of the DSHNHL [25]. To our knowledge, this is the largest pharmacogenomics study to explore the association of CEP72 rs924607 and VIPN in adult patients with aggressive B-cell lymphoma receiving (R-)CHOP based immunochemotherapy in the context of a prospective randomized phase III trial. Neuropathy was assessed systematically using the National Cancer Institute Common Toxicity Criteria ensuring the use of objective assessment criteria. On the other hand, neuropathy was assessed and reported by the treating physicians at the study sites without the use of a systematic objective assessment tool. This may even have led to an underreporting of VIPN in our patient cohort.

Our results could not confirm the data of Diouf et al. by failing to demonstrate an association of TT genotype of the CEP72 gene at rs924607 with VIPN in the entire analysed population. However, TT genotype was associated with incidence and severity of reported VIPN in female patients. Grade 1–4 and grade 2–4 VIPN was more common in females with TT genotype compared to women with CC or CT genotype. This difference was also observed for grade 3–4 VIPN (16% vs. 8%) but did not reach statistical significance, probably due to the small number of patients. To our knowledge, it is for the first time that sex-specific differences in VIPN are reported in a clinical patient cohort.

Sexual dimorphism in VIPN patterns has been previously demonstrated by separate groups in animal studies [26]. ^,^ [27]^,^ [28] Joseph et al. demonstrated that the severity of VCR-induced painful peripheral neuropathy in rats was sex-dependent [28]. Not only was neuropathy more prominent in female rats, but it was also abrogated with gonadectomy and reintroduced with estrogen replacement in females who had undergone gonadectomy. Thus, an estrogen-dependent sexual dimorphism could be demonstrated. Iguchi et al. examined the effects of vincristine in the autonomic and somatic nervous systems involved in lower urinary track function in mice [26]. Again, sex-dependent patterns of sensory neuropathy were demonstrated. Furthermore, gene expression analyses demonstrated increased CEP72 mRNA levels after VCR exposure in males but not in females. Whether the sexual dimorphism observed in the current study is a result of genetic or hormonal factors, cannot be clearly answered, particularly when considering, that all patients in the current study are almost certainly postmenopausal.

The fact that previous studies did not report sex-specific differences in VIPN can be attributed to at least two reasons. First, previous studies focused on children, in whom sex-hormone dependent effects do not yet come to bear [19]. ^,^ [22]^,^ [23] Second, studies focusing on adults receiving VCR included only small numbers of patients, making it likely that statistical power was not reached to reveal the influence of sex on VIPN [17, 20, 24]. 

Hence, when comparing VIPN in children and adults, different aspects have to be considered. The VCR dose that was administered within the RICOVER-60 trial was capped at 2 mg per cycle resulting in a cumulative dose of 12–16 mg. Children treated for ALL can receive cumulative doses of up to 47 mg/m² [19]. ^,^ [24] This significant dose difference makes comparisons between these patient groups more difficult.

Considering this, our results only partly support previous findings, i.e. homozygosity for CEP72 rs924607 is associated with an increased risk of VIPN, but provide evidence for the first time for the influence of sex on the development of VIPN. As consequence, female patients who are with the TT genotype might need closer monitoring regarding the development of VIPN with subsequent VCR dose reductions. Recent evidence suggests that VCR dose reductions are not associated with inferior outcome in patients with aggressive B-cell lymphomas [17]. On the other hand, the role of VCR in the standard treatment of B-cell lymphoma patients is currently being challenged. Polatuzumab vedotin seems to be an effective substitution for VCR in the treatment of patients with aggressive B-cell lymphoma as it has been shown to increase PFS in the phase III POLARIX trial, although it is currently not universally available [6]. Whether PNP induced by newer agents with a known neurotoxic side effect profile such as polatuzumab vedotin or brentuximab vedotin is also affected by polymorphisms in the CEP72 gene is an intriguing question for future pharmacogenomic analyses.

## Electronic supplementary material

Below is the link to the electronic supplementary material.

Supplementary Material 1

## Data Availability

The raw data can be made available upon request to the corresponding author.
