# Supplementary material for: An inherited genetic variant of the CEP72 gene is associated with the development of vincristine-induced peripheral neuropathy in female patients with aggressive B-cell lymphoma
Source: Ann Hematol. 2024 Sep 4;103(11):4599–606. doi: 10.1007/s00277-024-05973-9 (PMC11534822; doi:10.1007/s00277-024-05973-9)
Supplement: Supplementary file 1 — Supplementary Material 1 [file 277_2024_5973_MOESM1_ESM.docx]

**Supplementary data**

**Supplemental Figure 1: Flow Diagram of analyzed patients**

RICOVER-60

(n = 1222)

SNP rs924607

available

(n = 519)

Final Analysis

(n = 499)

Missing data for VIPN

(n = 20)

**Supplemental Figure 2: Incidence of PNP according to the presence and zygosity of rs924607 SNP**

Light green bars represent the percentage of patients with grade 1-4 PNP, medium shade green bars represent the percentage of patients with grade 2-4 PNP, and dark green bars represent the percentage of patients with grade 3-4 PNP. Incidence of PNP is shown for patients who are homozygous for the rs924607 SNP (right), patients heterozygous for the rs924607 SNP (middle) and patients with the wild type genotype (left)..

**Supplemental Figure 3: Sex-specific incidence of PNP according to presence and zygosity of rs924607 SNP**

Light green bars represent the percentage of patients with grade 1-4 PNP, medium shade green bars represent the percentage of patients with grade 2-4 PNP, and dark green bars represent the percentage of patients with grade 3-4 PNP. Incidence of PNP is shown for patients who are homozygous for the rs924607 SNP (right), patients heterozygous for the rs924607 SNP (middle) and patients with the wild type genotype (left), separately for male (left half) and female patients (right half).

**Supplemental Table 1. Comparison of baseline characteristics of patients with available germline DNA and available VIPN Data and the entire RICOVER-60 study cohort**

|  | Germline DNA + VIPN data available  (n = 499) | RICOVER-60  (n = 1222) | p-value** |
| --- | --- | --- | --- |
| Male  Female | 264 (53%)  235 (47%) | 650 (53%)  572 (47%) | 0.868 |
| Age, median (range) | 68 (61, 80) | 68 (61, 80) | 0.597 |
| LDH > UNV | 239 (48%) | 604 (49%) | 0.374 |
| ECOG > 1 | 63 (13%) | 176 (14%) | 0.142 |
| Stage III/ IV | 255 (51%) | 619 (51%) | 0.795 |
| Extralymph. inv. | 268 (54%) | 663 (54%) | 0.749 |
| Extralymph. inv. > 1 | 84 (17%) | 216 (18%) | 0.521 |
| IPI 1  2  3  4,5 | 158 (32%)  136 (27%)  127 (25%)  78 (16%) | 372 (30%)  339 (28%)  313 (26%)  198 (16%) | 0.882 |
| Bulky disease | 199 (40%) | 463 (38%) | 0.233 |
| B-symptoms | 166 (33%) | 399 (33%) | 0.703 |
| BM involvement | 26 (5%) | 73 (6%) | 0.350 |
| Reference pathology*  DLBCL  other B-cell  other | 407 (82%)  87 (18%)  3 (1%) | 949 (80%)  213 (18%)  29 (2%) | 0.002 |
| 6xCHOP-14  8xCHOP-14  6xCHOP-14 + 8xR  8xCHOP-14 + 8xR | 127 (25%)  118 (24%)  121 (24%)  133 (27%) | 307 (25%)  305 (25%)  306 (25%)  304 (25%) | -- |

* some missing values

** p-value for the comparison of available versus not available

Abbreviations: LDH = Lactatdehydrogenase; UNV = Upper Normal Value, ECOG = Eastern Cooperative Oncology Group performance status, extralymph. = extralymphatic; inv. = involvement; BM = bone marrow, DLBCL = diffuse large B-cell lymphoma
